# Comprehensive identification of RNA transcripts and construction of RNA network in chronic obstructive pulmonary disease

**DOI:** 10.1186/s12931-022-02069-8

**Published:** 2022-06-11

**Authors:** Pengcheng Liu, Yucong Wang, Ningning Zhang, Xiaomin Zhao, Renming Li, Yu Wang, Chen Chen, Dandan Wang, Xiaoming Zhang, Liang Chen, Dahai Zhao

**Affiliations:** 1 Department of Respiratory and Critical Care Medicine, The Second Affiliated Hospital, Anhui Medical University, 678 Furong Road, Hefei, 230601 Anhui Province China; 2 Department of Clinical Laboratory, The First Affiliated Hospital of USTC, The CAS Key Laboratory of Innate Immunity and Chronic Disease, School of Basic Medical Sciences, Division of Life Science and Medicine, University of Science and Technology of China, Hefei, 230027 China; 3 School of Basic Medicine, Anhui Medical University, Hefei, 230601 China

**Keywords:** COPD, circRNA, lncRNA, miRNA, RNA network

## Abstract

**Background:**

Chronic obstructive pulmonary disease (COPD) is one of the world’s leading causes of death and a major chronic disease, highly prevalent in the aging population exposed to tobacco smoke and airborne pollutants, which calls for early and useful biomolecular predictors. Roles of noncoding RNAs in COPD have been proposed, however, not many studies have systematically investigated the crosstalk among various transcripts in this context. The construction of RNA functional networks such as lncRNA-mRNA, and circRNA-miRNA-mRNA interaction networks could therefore facilitate our understanding of RNA interactions in COPD. Here, we identified the expression of RNA transcripts in RNA sequencing from COPD patients, and the potential RNA networks were further constructed.

**Methods:**

All fresh peripheral blood samples of three patients with COPD and three non-COPD patients were collected and examined for mRNA, miRNA, lncRNA, and circRNA expression followed by qRT-PCR validation. We also examined mRNA expression to enrich relevant biological pathways. lncRNA-mRNA coexpression network and circRNA-miRNA-mRNA network in COPD were constructed.

**Results:**

In this study, we have comprehensively identified and analyzed the differentially expressed mRNAs, lncRNAs, miRNAs, and circRNAs in peripheral blood of COPD patients with high-throughput RNA sequencing. 282 mRNAs, 146 lncRNAs, 85 miRNAs, and 81 circRNAs were differentially expressed. GSEA analysis showed that these differentially expressed RNAs correlate with several critical biological processes such as “ncRNA metabolic process”, “ncRNA processing”, “ribosome biogenesis”, “rRNAs metabolic process”, “tRNA metabolic process” and “tRNA processing”, which might be participating in the progression of COPD. RT-qPCR with more clinical COPD samples was used for the validation of some differentially expressed RNAs, and the results were in high accordance with the RNA sequencing. Given the putative regulatory function of lncRNAs and circRNAs, we have constructed the co-expression network between lncRNA and mRNA. To demonstrate the potential interactions between circRNAs and miRNAs, we have also constructed a competing endogenous RNA (ceRNA) network of differential expression circRNA-miRNA-mRNA in COPD.

**Conclusions:**

In this study, we have identified and analyzed the differentially expressed mRNAs, lncRNAs, miRNAs, and circRNAs, providing a systematic view of the differentially expressed RNA in the context of COPD. We have also constructed the lncRNA-mRNA co-expression network, and for the first time constructed the circRNA-miRNA-mRNA in COPD. This study reveals the RNA involvement and potential regulatory roles in COPD, and further uncovers the interactions among those RNAs, which will assist the pathological investigations of COPD and shed light on therapeutic targets exploration for COPD.

**Supplementary Information:**

The online version contains supplementary material available at 10.1186/s12931-022-02069-8.

## Introduction

Chronic obstructive pulmonary disease (COPD) is one of the world’s leading causes of death characterized by severe respiratory symptoms and airflow limitation [1]. With the increasing smoking rates in developing countries and the aging population in high-income countries, World Health Organization predicted that the prevalence rate of COPD will keep rising in the next 40 years and that the number of patients who die of COPD and related diseases will exceed 5.4 million per annum by 2060 [2]. At present, treatment of COPD mainly focuses on controlling symptoms and limiting disease progression, and the development of targeted therapeutic drugs is slow [3]. Genetic basis of COPD remains elucidated, with specific and effective biomolecules for COPD early clinical prevention and treatments are still lacking [4].

Dysregulation of gene expression is a universal theme of human diseases, and the resulting abnormal mRNAs and non-coding RNAs play important roles in the occurrence and progression of various diseases, including COPD [5]. Next-generation sequencing facilitates our understanding of differentially expressed RNA and its significance in COPD. For example, 82 gene locus were found to be associated with COPD through a genome-wide association study (GWAS), suggesting a genetic basis for the clinical heterogeneity seen in COPD [6]. Peripheral blood could also be used to capture relevant lung pathobiology through RNA-seq profiling [7]. A series of COPD-related genes have also been identified in gene expression profiling, with more comprehensive assessments of rare genetic variations are increasingly being used in COPD genetic research [8].

MiRNAs are critical regulators involved in multiple physiological and pathological processes, including development, organogenesis, apoptosis, and cell proliferation and differentiation [9]. Dysregulation of miRNA often leads to impaired cellular function and disease [10]. MicroRNA-218 acts by inhibiting TNFR1-mediated activation of NF-κB, which is associated with the overproduction of MUC5AC and inflammation in smoking-induced bronchiolitis of COPD [11]. In terms of treatment, through bronchial biopsies of 69 moderate/severe COPD patients and related in vitro experiments, miR-320d inhibits the expression of inflammatory cytokines and regulates the pro-inflammatory response of the airway epithelium in patients with COPD as a novel mediator of Inhaled Corticosteroids (ICS) [12].

LncRNAs are widely involved in various biological processes, such as regulation of transcription, RNA splicing, etc. [13]. Aberrant expressions of lncRNA are closely related to various human diseases. For instance, SIRT1/p53 and FOXO3a signaling pathways mediated by senescence-associated lncRNA1 (SAL-RNA1) regulate the expression of SIRT1 and FOXO3a, promoting the senescence of type II alveolar epithelial cells (AECII) in the pathogenesis of COPD [14]. In a microarray study of genome-wide lncRNA expression profiling in lung tissue of patients with COPD, 120 lncRNAs were up-regulated and 43 lncRNAs were down-regulated in the lung tissue of COPD patients compared with normal smokers without COPD [15].

CircRNAs are a special class of non-coding RNAs with a covalently-closed loop generally through 5’ to 3’ ends back splicing in animals, involved in various diseases including COPD. circRNAs play a significant role in regulating gene expression by binding to specific microRNAs or RNA binding proteins. For instance, hsa_Circ_0016070 promotes the proliferation of pulmonary artery smooth muscle cells (PASMCs) and participates in vascular remodeling in pulmonary hypertension (PAH) via the miR-942/CCND1 axis [16]. Epithelial–mesenchymal transition (EMT) may promote small airway remodeling and fibrosis in patients with COPD and increase the likelihood of lung cancer [17], circ0061052 regulates the expression of FoxC1/Snail by competitively binding miR-515-5p in human bronchial epithelial cells and involves in the CS-induced EMT and airway remodeling in COPD [18].

In this study, we have comprehensively identified differentially expressed mRNAs, miRNAs, lncRNAs, and circRNAs in the peripheral blood of COPD patients through high-throughput RNA sequencing. GSEA analysis showed that these differentially expressed RNAs correlate with several RNA biological processes such as ncRNA metabolic process, ncRNA processing, ribosome biogenesis, rRNA metabolic process, tRNA metabolic process, and tRNA processing. To demonstrate the potential interactions between lncRNAs and their interacting mRNAs, we have constructed the lncRNA-mRNA co-expression network in COPD. For the validated circRNAs, we have analyzed the features of the circRNAs and predicted their potential functional mechanisms in COPD. We further for the first time constructed the circRNA-miRNA-mRNA network in COPD, which provides a valuable prognostic or predictive resource for future COPD research and clinical treatments.

## Methods

### Clinical samples

All fresh peripheral blood samples of three patients with COPD and three non-COPD patients from the Second Affiliated Hospital of Anhui Medical University, which was approved by the Ethics Committee of the Second Affiliated Hospital of Anhui Medical University [YX2021-148(F1)]. Written informed consent was obtained from each patient for this study. Information of the patients is included in Tables 1 and 2.

**Table 1:** Information of the patients for RNA-seq

Characteristics	Control group (n = 3)	COPD group (n = 3)	p-value
Male gender	3 (100.00)	3 (100.00)	> 0.999
Age, years	59.00 ± 4.32	58.00 ± 4.55	0.833
BMI, kg/m^2^	23.51 ± 1.82	25.78 ± 3.34	0.446
Ever smoker	0 (100.00)	3 (100.00)	0.100
Current smoker	0 (100.00)	3 (100.00)	0.100
Smoking history (pack-years)	0 ± 0.00	41.67 ± 13.12	0.047
FEV_1_ (% predicted)	112.40 ± 16.03	55.70 ± 4.65	0.009
FEV_1_/FVC (%)	82.16 ± 6.33	58.84 (7.69)	0.030
CAT score	0 ± 0.00	6.67 ± 5.25	0.214

Data are presented as mean (SD) or number (percentage); The difference between the two groups was analyzed by independent-sample t-test and One-way ANOVA

*BMI* body mass index, *FEV1* forced expiratory volume in 1 s,* FVC* forced vital capacity

**Table 2:** Information of the patients for RT-qPCR validation

Characteristics	Control group (n = 27)	COPD group (n = 24)	p-value
Male gender	20 (74.1)	19 (79.2)	0.749
Age, years	63.44 ± 10.73	66.21 ± 9.38	0.344
BMI, kg/m^2^	23.19 ± 3.02	23.79 ± 3.26	0.504
Ever smoker	7 (25.9)	13 (54.2)	0.049
Current smoker	5 (18.50)	8 (33.30)	0.336
Smoking history (pack-years)	41.43 ± 15.29	44.23 ± 17.19	0.736
FEV1 (%)	107.31 ± 17.23	51.19 ± 18.03	< 0.001
FEV1/FVC (%)	80.63 ± 5.54	48.52 ± 11.23	< 0.001
CAT Score	0 ± 0.00	7.96 ± 7.73	< 0.001

Data are presented as mean (SD) or number (percentage); The difference between the two groups was analyzed by independent-sample t-test and One-way ANOVA

*BMI* body mass index, *FEV1* forced expiratory volume in 1 s,* FVC* forced vital capacity

### Total RNA extraction

The peripheral blood samples and TRizol reagent (Invitrogen, USA) are evenly mixed in a ratio of 1:3, a total of 12 ml. Total RNA was extracted by using TRizol reagent according to the manufacturer’s instructions.

### Library construction and high-throughput sequencing

Total RNAs from peripheral blood of three patients with COPD and their corresponding control patients without COPD were extracted for high-throughput sequencing. Whole transcriptome libraries were constructed by the TruSeq Ribo Profile Library Prep Kit (IL, USA), according to the manufacturer’s instructions. In brief, 10 mg total RNA was depleted rRNA with an Illumina Ribo-Zero Gold kit and purified for end repair and 50-adaptor ligation. Then, reverse transcription was performed with random primers containing 30 adaptor sequences and randomized hexamers. Finally, the cDNAs were purified and amplified with a thermo cycler. The PCR products of 300–500 bp were purified, quantified, and stored at -80℃ before sequencing. The libraries were subjected to 150 nt paired-end sequencing with Illumina novaseq 6000.

### Transcriptome sequencing data analysis

To analyze mRNAs and lncRNAs, the high-throughput sequencing tools, Hisat2, and feature_counts, were used to map clean reads to *Homo* sapiens reference genome (hg19) and calculate the gene expression level which was normalized to FPKM. To determine the differentially expressed mRNA and lncRNA, the “DEseq2” package in R software was used with the corresponding cutoff (q < 0.05, |log2(fold change)|> 1 for mRNA and lncRNA). To predict circular RNA (circRNA), we identified the possible candidates with find_circ, the junction reads were normalized to TPM. Standard of P < 0.05, and|log2(fold change)|> 1 was used to identify differentially expressed circRNAs. The prediction of microRNA response element (MRE) and RNA binding protein (RBP) among differentially expressed circRNAs were analyzed by the CircRNA database (CSCD) (http://gb.whu.edu.cn/CSCD/).

### Small RNA data analysis

For small RNA (sRNA) sequencing, six sRNA libraries were generated with TruSeq small RNA (IL, USA) according to the manufacturer’s instructions. Then the prepared libraries were sequenced with an Illumina novaseq 6000. After filtering out the reads shorter than 15 nt, the remaining reads were mapped to the human genome (hg19) and the miRNA database in miRBase with bowtie (− v 1). The differentially expressed miRNAs were determined by DEseq2 with the cutoff of P < 0.1, |log2(fold change)|≧1.

### Construction of lncRNA-mRNA co-expression network

To construct co-expression network between significantly differentially expressed lncRNA and mRNA. Firstly, the Pearson’s correlation coefficients between lncRNA and mRNA were calculated by cor function in R software. A criterion of the coefficient parameter |R|> 0.99 was used for the remaining RNAs to further construct the network. For visualization of the network, the Cytoscape software (https://cytoscape.org, v3.4.0) was used.

### Construction of circRNA-miRNA-mRNA network

To construct a circRNA-miRNA-mRNA network between differentially expressed circRNA, miRNA, and mRNA. Firstly, 6 up-regulated circRNAs were verified by RT-qPCR. Next, the miRanda (http://www.microrna.org/microrna/home.do) and TargetScan (http://www.targetscan.org/vert_72/) were used to identify miRNAs targeting on these circRNAs. The miRDB (http://www.mirdb.org/) and miRTarBase (http://miRTarBase.mbc.nctu.edu.tw/) were applied to predict the target mRNAs of miRNAs. Then, the Pearson correlation coefficient (R) in these interactions were calculated by the cor function in R software. These correlation coefficient were used to estimate the co-expression relationship between circRNAs, mRNAs, and miRNAs. The strong interactions between miRNAs, circRNAs, and mRNAs (with a threshold of |R|> 0.99) were selected to construct a circRNA-miRNA-mRNA network using Cytoscape (https://cytoscape.org, v3.4.0).

### Gene set enrichment analysis (GSEA)

The GSEA analysis was performed to annotate the function of protein-coding genes (PCGs) in the peripheral blood of COPD patients. These PCGs were pre-ranked and the GSEA analysis was implemented in GSEA software (version 4.1.0, https://www.gsea-msigdb.org).

### Reverse transcription and real-time quantitative PCR

cDNA was synthesized using the GoScript Reverse Transcription System (Promega, USA) according to the manufacturer’s protocol. Quantitative real-time PCR was performed with GoTaq SYBR Green qPCR Master Mix (Promega, USA) on a PikoReal 96 real-time PCR system followed by 40 amplification cycles (Thermo Fisher Scientific, USA) according to standard procedures. Actually, all amplification curves already reached the stationary stage before 35 amplification cycles, and the Ct value was obtained at the exponential stage. Relative RNA level was normalized to GAPDH mRNA. All primers are shown in Additional file 3: Table S1.

### Statistical analysis

The nortest (v 1.0.4) R package was used to assess whether the data follows normal distribution in all experiments. Student’s *t*-tests were used to calculate P-values by t.test function in R software. The values reported in the graphs represent average of actual number of independent experiments, with error bars showing SD. The cor function in R software was used to calculate correlation coefficients of all groups. The cluster analysis in heatmaps was conducted by cluster (v 2.1.2) R package. The heatmaps were generated by pheatmap R package (v 1.0.12). The bar plots and scatter plots were generated by ggplot2 (v 3.3.3). After analysis of variance with F-tests with R software, the statistical significance and P-values were evaluated with Student’s *t*-tests.

## Results

### Identification of differentially expressed mRNAs and lncRNAs in COPD

**Fig. 1:**
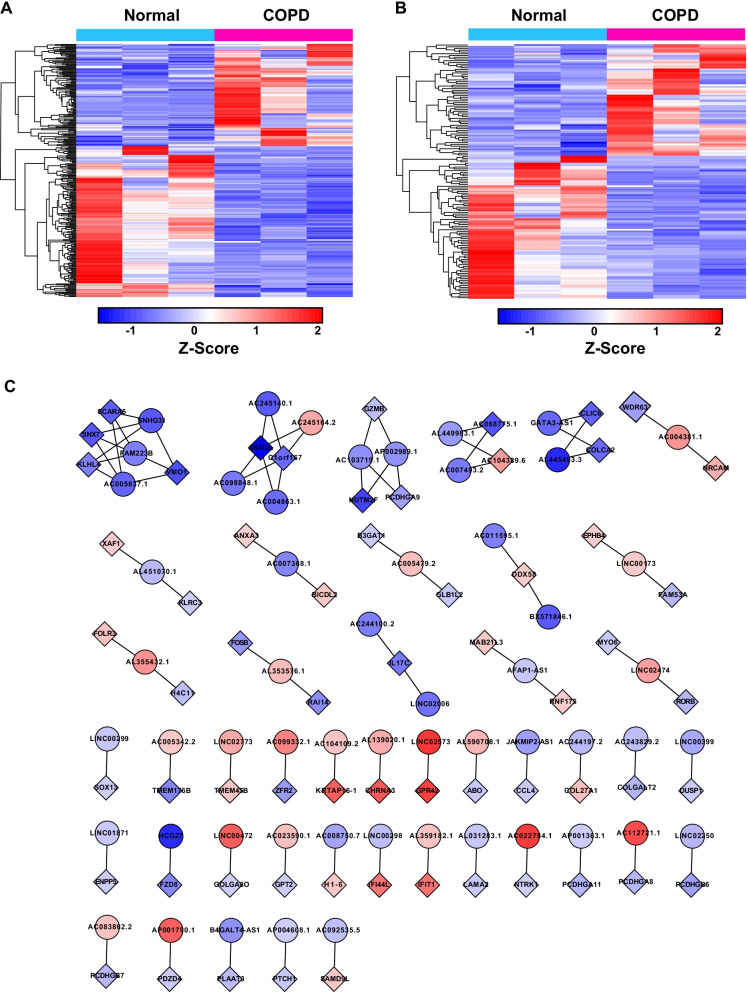
Analytical procedure and the analysis of mRNA and lncRNA in COPD patients and healthy individuals. The analytical procedure of COPD patient and health individual. **A** Hierarchical cluster heatmaps display differentially expressed transcripts among mRNA (**B**) and lncRNA (**C**). **D** The co-expression network between differentially expressed lncRNA and mRNA (correlation coefficient absolute value > 0.99). The boxes represent mRNA, the circles represent lncRNA. Red, upregulated; blue, downregulated

To identify COPD-related RNAs, we used fresh peripheral blood samples from COPD patients for RNA sequencing. Fresh peripheral blood from three patients without COPD was used as controls (Fig. 1A). Patient characteristics are shown in Table 1. All the COPD patients were clinically stable without any acute infection and were not on corticosteroid therapy. RNA-seq data of sufficient quality was obtained for mRNA, lncRNA, miRNA, and circRNA expression profiles. We first set out to more specifically investigate the differentially expressed mRNAs and lncRNAs with filtration criteria (fold changes ≥ 2.0 and p-values ≤ 0.05) (Fig. 1B, C). We identified 282 mRNAs and 146 lncRNAs that were differentially expressed in COPD (Additional file 4: Table S2, Additional file 5: Table S3). Among them, 115 mRNAs were upregulated, 167 mRNAs were downregulated; 64 lncRNAs were upregulated and 82 lncRNAs were downregulated in COPD. Accumulative evidence suggests that lncRNAs can interact with co-expressed mRNAs through the formation of complementary hybrids, working from both nearby and distant sites. We, therefore, constructed the lncRNA-mRNA co-expression to reveal the co-regulatory relationship network of COPD based on their potential multi-reciprocal interactions (Fig. 1D). We performed the correlation analysis for mRNAs and lncRNAs by calculating the Pearson correlation coefficient in all samples and selected lncRNA-mRNA pairs with |R|> 0.99 for co-expression network construction. Of these interactions, most coding genes are associated with distinct lncRNAs. A few coding genes were regulated by multiple lncRNAs, such as SCARA5, SNX7, KLHL4, VMO1, SKIV2L, C1orf167, GZMB, NUTM2F, PCDHGA9, AC068775.1, AC104389.6, CLIC6, and COLCA2. These lncRNA-mRNA co-expression networks provided the potential functional interactions of lncRNA-mRNA for future studies.

### GSEA analysis of the differentially expressed mRNAs

**Fig. 2:**
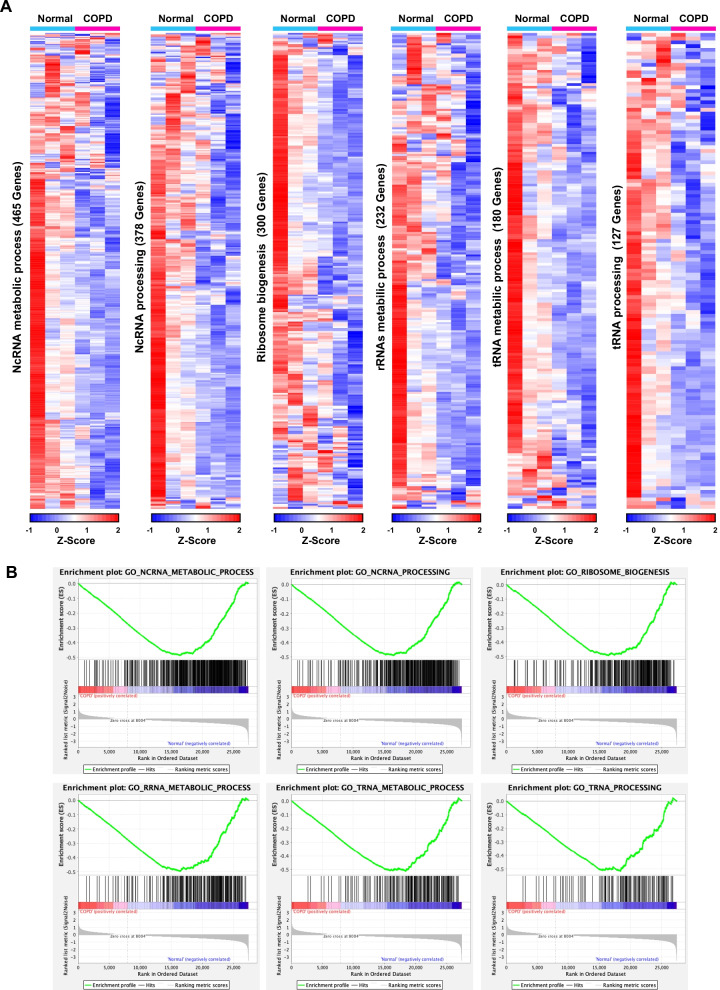
GSEA analysis from COPD RNA-seq. **A** Heatmap of gene expression level in six GO terms related to RNA processes. **B** Enrichment plot of above six GO ontologies among RNA processes

To investigate the potential RNA-related pathways and biological processes that these differentially expressed mRNAs involved in COPD, we performed GSEA analysis with either upregulated or downregulated mRNAs (Fig. 2). Of 3972 gene sets, 3756 gene sets (94.56%) were downregulated in the COPD group compared to the normal group. 444 gene sets are significantly enriched at FDR < 25%, and 396 gene sets are significantly enriched at nominal p-value ≤ 1% (Additional file 8: Tables S6, Additional file 9: Table S7). According to KEGG pathway enrichment analysis, downregulated gene sets in COPD were most enriched in six pathways: “ncRNA metabolic process”, “ncRNA processing”, “ribosome biogenesis”, “rRNAs metabolic process”, “tRNA metabolic process” and “tRNA processing”, all of which were downregulated in COPD group. 465 genes were enriched in “ncRNA metabolic process”, among which the top five genes correlated with COPD were TDRD1, LYAR, HENMT1, TRIM71, and SLFN13; 378 genes were enriched in “ncRNA processing”, among which the top five genes correlated with the COPD were LYAR, TSEN54, HSD17B10, ELAC1, and NSA2; 300 genes were enriched in “ribosome biogenesis”, among which the top five genes correlated with the COPD were LYAR, MRPL11, RSL24D1, NSA2, and TSR2; 232 genes were enriched in “rRNAs metabolic process”, among which the top five genes correlated with the COPD were LYAR, SLFN13, NSA2, TSR2, and NHP2; 180 genes were enriched in “tRNA metabolic process”, among which the top five genes correlated with the COPD were SLFN13, TSEN54, HSD17B10, ELAC1 and TARS1; 127 genes were enriched in “tRNA processing”, among which the top five genes correlated with the COPD were TSEN54, HSD17B10, ELAC1, TRMT61B, and OSGEPL1.

### Validation of differentially expressed mRNA and lncRNAs in clinical samples

**Fig. 3:**
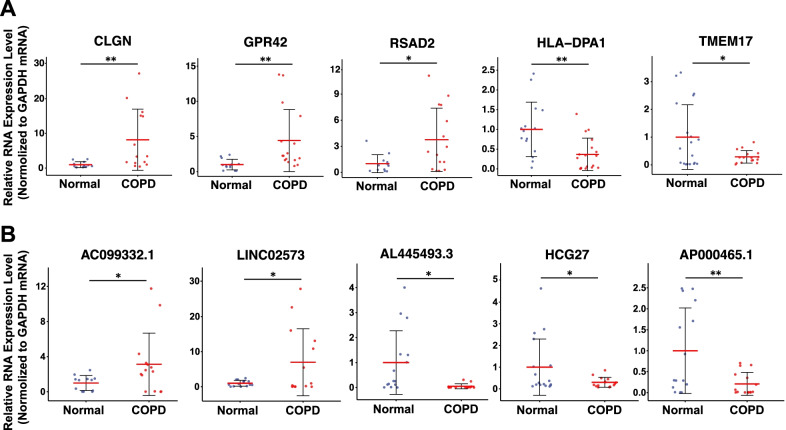
Validation of mRNAs and lncRNAs identified from RNA-seq data by RT-qPCR. The validation results of (**A**) differentially expressed mRNAs, and (**B**) differentially expressed lncRNAs. Data are median SD. *p < 0.05, **p < 0.01 are based on the Student’s *t-*test

To validate the differentially expressed mRNAs and lncRNAs from the COPD RNA-seq, we randomly chose the differentially expressed mRNAs and lncRNAs respectively for RT-qPCR validation with COPD patient sample pairs (Fig. 3). The housekeeping gene GAPDH was used as the endogenous control. Patient characteristics are shown in Table 2. In the mRNA group, CLGN, GPR42, and RSAD2 were significantly upregulated in the blood of COPD patients compared to the normal controls; HLA-DPA1 and TMEM17 were significantly downregulated compared to the normal control (Fig. 3A). In the lncRNA group, AC099332.1 and LINC02573 was significantly upregulated in COPD patients, while AL445493.3, HCG27, and AP000465.1 were significantly downregulated in COPD patients, compared to the normal controls (Fig. 3B). The present results showed that the RT-qPCR verification was in accordance with RNA-seq results with high confidence.

### Analysis of the differentially expressed miRNAs and circRNAs in COPD

**Fig. 4:**
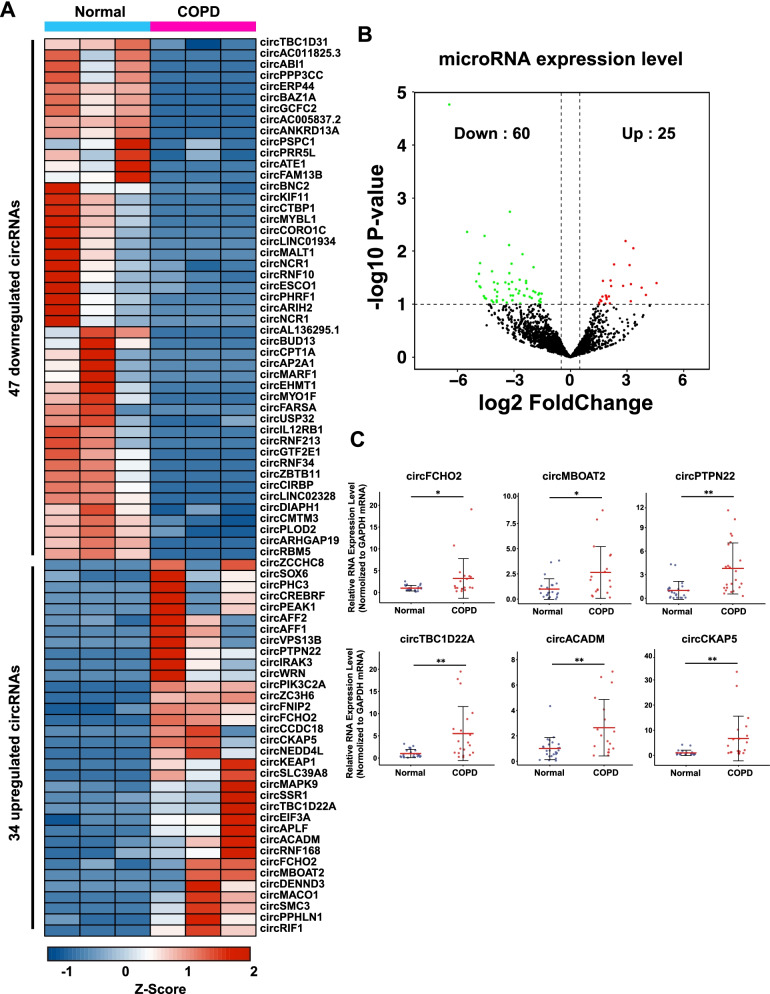
The analysis of circRNA and miRNA in COPD. **A** Heatmap display of differentially expressed circRNAs. **B** Volcano plot display differentially expressed transcripts of miRNAs. **C** Validation of 6 differentially expressed circRNAs by RT-qPCR

CircRNAs and miRNAs, as well as their functional interactions, play vital roles in the regulation of many biological processes including COPD. We then set out to analyze the differentially expressed circRNAs and miRNAs with filtration criteria (circRNA: fold changes ≥ 2.0 and p values < 0.05; miRNA: fold changes ≥ 2.0 and p values < 0.1) (Fig. 4A, B) [19–21]. 85 miRNAs were differentially expressed, with 60 miRNAs (70.59%) were downregulated, while 25 miRNAs (29.41%) were upregulated (Additional file 6: Table S4). Totally 81 circRNAs were differentially expressed, and a heatmap was performed to show either upregulated or downregulated circRNAs. 47 circRNAs (58.02%) were downregulated, while 34 circRNAs (41.98%) were upregulated (Additional file 7: Table S5). To validate the differentially expressed circRNAs from COPD RNA-seq, we chose 6 differentially expressed circRNAs for RT-qPCR validation with COPD patient sample pairs (Fig. 4C). The housekeeping gene GAPDH was used as the endogenous control. Patient characteristics are shown in Table 2. CircFCHO2, circMBOAT2, circPTPN22, circTBC1D22A, cirCOPDADM, and circCKAP5 were significantly upregulated in the blood of COPD patients compared to the normal control, which was all in accordance with RNA-seq results with high confidence.

### Construction of circRNA-miRNA-mRNA network of COPD

**Fig. 5:**
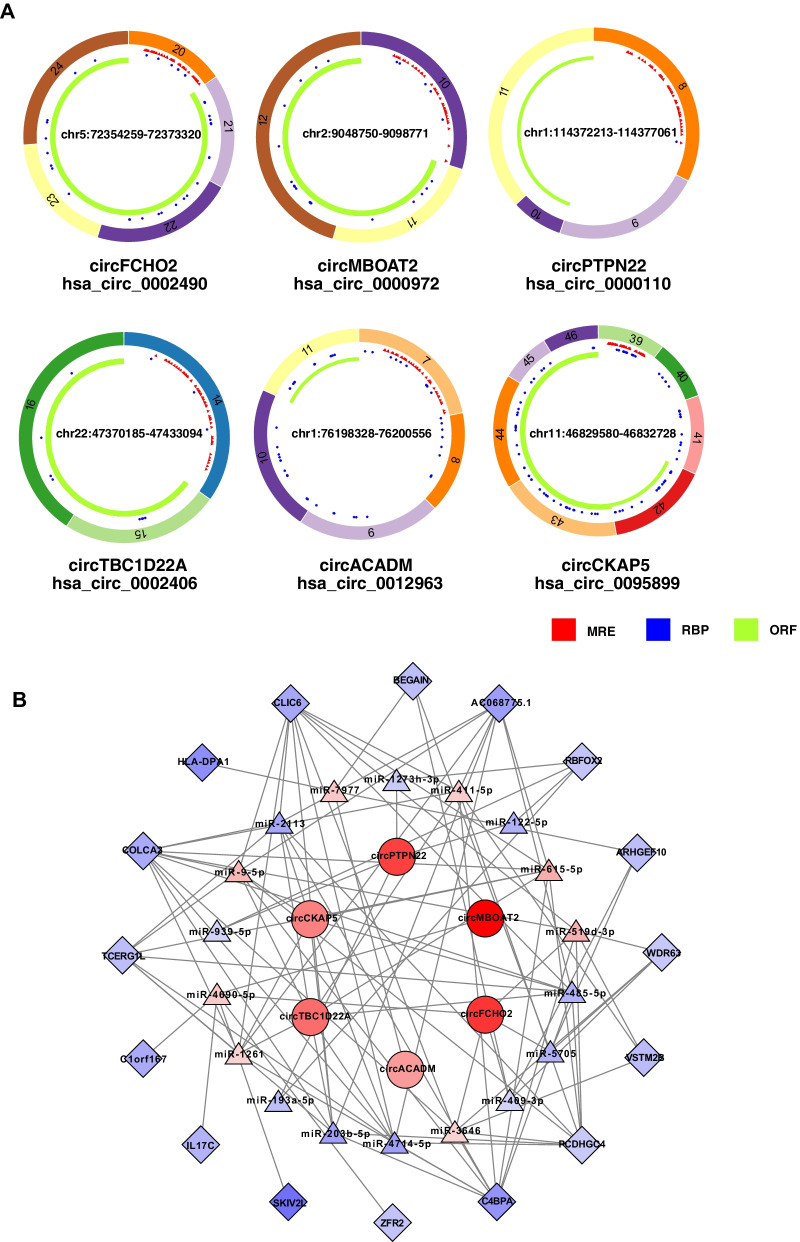
Prediction of the functional mechanism of differentially expressed circRNAs and their functional network in COPD. **A** Functional mechanism prediction of 6 differentially expressed circRNAs. MRE, miRNA response elements. RBP, RNA binding protein. ORF, open reading frame. **B** Construction of circRNA-miRNA-mRNA network for 6 differentially expressedcircRNAs according to the interactions among circRNAs, mRNAs, and miRNAs (correlation coefficient absolute value > 0.99)

CircRNAs participate in a variety of biological processes through multiple mechanisms due to their unique structures and properties. We then investigated the above 6 validated circRNAs based on their structures, and performed functional prediction (Fig. 5A). Three criteria for circRNAs were mainly focused: MRE (MicroRNA Element) for potential miRNA binding capabilities; RBP (RNA Binding Protein) for circRNA-RBP interaction; ORF (Open Reading Frame) for circRNA as a potentially translatable template [22–26]. CircRNAs with miRNA binding sites could bind directly to the corresponding miRNAs to inhibit miRNA activity and thus regulate the expression of target genes [22, 27]. All the 6 circRNAs have miRNA binding sites, indicating they might function through miRNA sponging, which is also the most reported circRNA function. CircRNAs can act as scaffolds or decoys of RBPs, and thus exert their functions through interactions with the RBPs. All the circRNAs examined but circPTPN22 have predicted RBP binding sites, suggesting they might function as RNA-binding protein scaffold. Some circRNAs could act as translation templates, and their polypeptide products have been shown to play roles in physiology and disease. All the circRNAs have ORF, which indicated that they possessed capability for translation and may serve as functional polypeptides. Of note, circRNAs might function through one or multiple mechanisms depending on specific interacting factors and/or various contexts, which need further investigations and verifications for the predicted mechanisms.

To investigate the potential function of circRNAs acting as miRNA sponges in COPD, we hence constructed a circRNA-miRNA-mRNA network based on our validated 6 circRNAs and their corresponding regulating miRNAs and mRNAs in COPD. 18 miRNAs and 16 mRNAs were found to be correlated with this ceRNA network (Fig. 5B; Additional file 10: Table S8), which indicates that these identified six circRNAs might function through interaction directly or indirectly with these miRNAs and mRNAs.

## Discussion

COPD is a major heterogeneous disease, which becomes one of the leading causes of death worldwide[28]. Bronchodilators and anti-inflammatory agents provide the major treatment for COPD patients, however, with poor treatment outcome. Better understandings of the pathophysiology of disease development are recognized to be of increasing clinical importance, and identification of critical therapeutic targets specifically for COPD is of utmost importance [29]. RNA therapeutics provide high specificity and represent safer and reversible alternatives to DNA-based gene therapies, and RNA drugs potentially offer unique opportunities to expand the range of therapeutic targets with several of them have been approved or being currently in clinical trials [30]. This study uses RNA-seq to preliminarily explore the differentially expressed mRNAs, lncRNAs, miRNAs and circRNAs during the process of COPD, and tentatively reveal the interactions and potential functional mechanisms of these RNAs. We hypothesized that certain dysregulated RNAs could exert their functions either individually or cooperatively, and some of them could be exploited as potential biomarkers.

High-throughput sequencing technologies have facilitated the detection of aberrantly expressed both protein-coding and noncoding genes in humans at the transcriptome level [31]. The number of studies focusing on the disease-related RNAs is fast increasing as differential expression of specific genes would positively or negatively correlate with disease pathology. Increasing evidence long suggests that lncRNAs, miRNAs, and circRNAs along with mRNAs could massively involved in the progression of many diseases, with some of them being identified as potentially suitable biomarkers [32, 33]. However, there is only a limited number of studies for the universal investigations on the differentially expressed RNAs of COPD, especially circRNAs [34].

In this study, we utilized RNA deep-sequencing from peripheral blood of COPD patients and identified 282 mRNAs, 146 lncRNAs, 85 miRNAs, and 80 circRNAs that were differentially expressed. To the best of our knowledge, it is the first comprehensive study that identifies and analyzes the differentially expressed mRNA, miRNA, lncRNA, and circRNA in COPD. We also validated some of the differentially expressedRNAs which were in accordance with the RNA-seq with high confidence. We also performed GSEA analysis with either upregulated or downregulated differentially expressed RNAs. Several critical pathways that associate with COPD were found in the analysis, especially noncoding RNA metabolic processing, RNA processing, ribosome biogenesis, rRNA metabolic process, tRNA metabolic process, and tRNA processing. It is acknowledged that these biological processes might be correlated with the progression of COPD, yet with limited evidence. For example, m^6^A RNA methylation regulators contribute to COPD progression, and the expressions of m6A RNA methylation regulators (IGF2BP3, FTO, METTL3, and YTHDC2) [35], which have significant associations with some key genes enriched in the signaling pathway and biological processes that promote the development progression of COPD, are highly correlated with the occurrence of COPD [36, 37]. Of note, six mRNAs (LYAR, SLFN13, TSEN54, HSD17B10, ELAC1, NSA2) listed in the six pathways of GESA analysis were significantly downregulated in at least three pathways, in which LYAR, TSEN54, and ELAC1 were enriched in at least four pathways. LYAR is a cell growth-regulating nucleolar protein, which is reported to potentiate rRNA synthesis and is important for influenza A virus replication [38]. LYAR is also found down-regulated proteins in well-differentiated normal human primary bronchial/tracheal epithelial cells compared with undifferentiated cells [39]. TSEN54 is a putative causal gene for lung function and is associated with the genetics of smoking behavior, lung function, and COPD (even in nonsmokers) [40]. Further studies are worthy of investigating whether the differentially expressed RNAs function in these biological processes [41]. In addition, we also found pathways known to be closely related to COPD pathology in the GSEA analysis, such as oxidative phosphorylation, protein folding, cytolysis, amino acid activation, rRNA transcription, ribosomal large subunit biogenesis [42–47] (Fig. S2).

lncRNA-mRNA interaction is one of the most frequently studied functional mechanisms of lncRNA [48]. We also constructed a lncRNA-mRNA co-expression network in COPD, based on the ceRNA mechanism which is similar to circRNA-miRNA interaction. It is long acknowledged that the RNA-seq-based lncRNA-mRNA network has become a useful tool to predict functional lncRNAs and their potential functional mechanisms, which is widely used in many disease models and clinical samples [49]. Our lncRNA-mRNA network in COPD would hopefully provide useful information for future COPD investigations.

Lines of evidence reveal that circRNAs participate in various biological processes, including cell proliferation, protein metabolism, autophagy, tumor immunology, signal transduction, genome stability, etc. [29, 50]. In this study, we presented 6 circRNAs that were also validated in the following experiments, and we demonstrated their potential mechanisms according to their nucleotide sequences. CircRNAs are generally found to function with multiple mechanisms, among them, the three main functions are miRNA sponge, protein scaffold, transcriptional regulation, and translation [51]. Of note, circRNA acting as miRNA sponge was first found in 2013 and has become one of the most investigated mechanisms in almost all disease models [50]. It is believed that circRNA, which harbors miRNA binding sites, would sequester miRNA(s) like a sponge and thus alleviate miRNA’s suppression of its target protein(s) [16]. Some circRNAs can act as RBP scaffolds or decoys to exert their function through RNA-RBP interaction. Also, some endogenous circRNAs can be used as scaffolds to regulate protein–protein interactions. Some other circRNAs can initiate peptide translation through internal ribosomal entry sites (IRES) or N6-methyladenosine (m6A) [52, 53]. Based on this, we first constructed the circRNA-miRNA-mRNA network in COPD, and 6 circRNAs, 18 miRNAs, 16 mRNAs were potentially correlated and were involved in the occurrence and progression of COPD, which indicates potential biological or clinical mechanisms for future studies. However, there is a certain possibility that these circRNAs might functionally interact with proteins or translate to polypeptides in other contexts due to the cell- and tissue-specificity of circRNAs [51]. Together with the lncRNA-mRNA co-expression network, we believe that a comprehensive understanding of the complex networks of interactions that these differentially expressed RNAs coordinate would provide a unique opportunity for better therapeutic interventions.

The findings of the study reveal potentially important RNAs, pathways and RNA networks that are associated with COPD, and provide a prospective view towards the underlying functions and functional mechanisms of these differentially expressed RNAs. Compared to the previous work in COPD, we have first conducted comprehensive bioinformatics analysis towards RNAs, especially circRNA, an emerging RNA molecule with unique criteria and clinical potential. To date, few studies have investigated the differential expression of multiple RNAs in COPD context, and this study provides valuable RNA resources for future in-depth COPD investigations. We have also explored the functional mechanisms based on these analyzed RNAs, and most importantly, established the interaction networks, especially the circRNA-miRNA-mRNA network in COPD, which provides sufficient novelty to this study.

However, some further questions remain that require future investigations. First, more COPD samples are expected for our RNA sequencing and bioinformatics analysis based on sample size estimation, which will optimize the study design for differential expression detection with high confidence. Second, it should be noted that we used peripheral blood from COPD patients for RNA-seq rather than bronchus due to the lack of these samples in clinical practices. The direct associations of the RNAs analyzed and COPD should be critically investigated in the future study.Third, the RNA regulatory networks were only based on bioinformatics predictions and lack practical experiments for verification which requires future in-depth studies with in vivo and in vitro experiments. The use of COPD animal models is also a useful tool in COPD investigations, which would facilitate the research on potential functions and evolutionary conservation of the RNAs. Our team is currently committed to studying these potential functions and mechanisms.

## Supplementary Information

**Additional file 1: Fig. S1. **Prediction of the functional mechanism of differentially expressed circRNAs. Functional mechanism prediction of 6 differentially expressed circRNAs. MRE, miRNA response elements. RBP, RNA binding protein. ORF, open reading frame.

**Additional file 2: Fig. S2**. GSEA analysis related to COPD from RNA-seq. Enrichment plot of six GO terms associated with COPD pathology, including oxidative phosphorylation, protein folding, cytolysis, amino acid activation, rRNA transcription, ribosomal large subunit biogenesis.

**Additional file 3: Table S1**. Primers used in this study.

**Additional file 4.** Differentially expressed mRNAs in this study.

**Additional file 5.** Differentially expressed lncRNAs in this study.

**Additional file 6.** Differentially expressed microRNAs in this study.

**Additional file 7.** Differentially expressed circRNAs in this study.

**Additional file 8.** 444 gene sets significantly enriched at FDR<25% in GSEA analysis.

**Additional file 9.** 396 gene sets significantly enriched at nominal p-value ≤1% in GSEA analysis.

**Additional file 10.** 18 miRNAs and 16 mRNAs in circRNA-miRNA-mRNA network.

## Data Availability

The datasets generated and analyzed in the current study are available in the GEO (Gene Expression Omnibus). The accession number is GEO: GSE198740.

## Abbreviations

ceRNA: Competing endogenous RNAs
circRNA: Circular RNA
COPD: Chronic obstructive pulmonary disease
FEV1: Forced expiratory volume in 1 s
FVC: Forced vital capacity
GSEA: Gene set enrichment analysis
lncRNA: Long non-coding RNA
miRNA: MicroRNA
mRNA: Message RNA
ncRNA: Non-coding RNA
qRT-PCR: Quantitative real-time PCR
RNA-seq: RNA-sequencing RNA
