# Supplementary material for: Comprehensive identification of RNA transcripts and construction of RNA network in chronic obstructive pulmonary disease
Source: Respir Res. 2022 Jun 11;23:154. doi: 10.1186/s12931-022-02069-8 (PMC9188256; doi:10.1186/s12931-022-02069-8)
Supplement: Supplementary file 1 — Additional file 1: Fig. S1. Prediction of the functional mechanism of differentially expressed circRNAs. Functional mechanism prediction of 6 differentially expressed circRNAs. MRE, miRNA response elements. RBP, RNA binding protein. ORF, open reading frame. [file 12931_2022_2069_MOESM1_ESM.pdf]

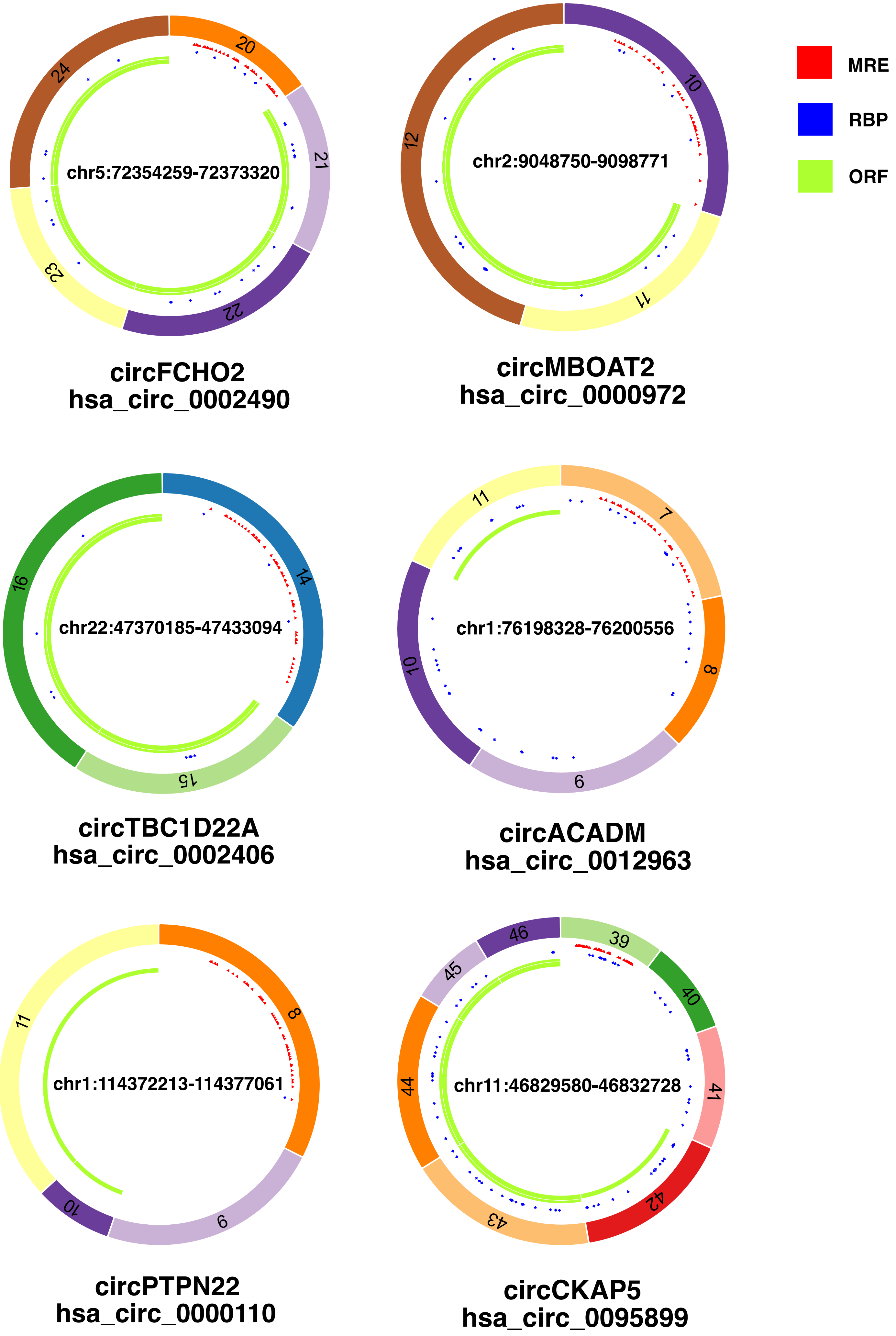
MRE
RBP
ORF
chr5:72354259-72373320
circFCHO2
hsa_circ_0002490
chr2:9048750-9098771
circMBOAT2
hsa_circ_0000972
chr22:47370185-47433094
circTBC1D22A
hsa_circ_0002406
chr1:76198328-76200556
circACADM
hsa_circ_0012963
chr1:114372213-114377061
circPTPN22
hsa_circ_0000110
chr11:46829580-46832728
circCKAP5
hsa_circ_0095899
