# Supplementary figures and images for: Comprehensive identification of RNA transcripts and construction of RNA network in chronic obstructive pulmonary disease
Source: Respir Res. 2022 Jun 11;23:154. doi: 10.1186/s12931-022-02069-8 (PMC9188256; doi:10.1186/s12931-022-02069-8)

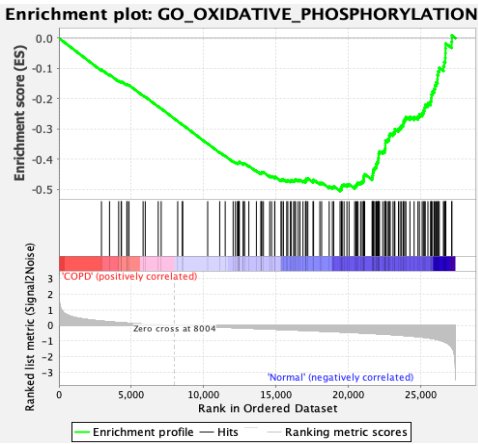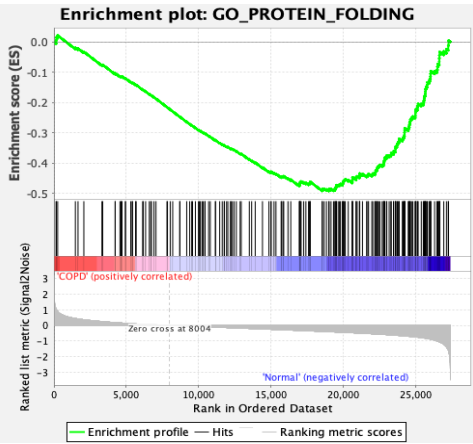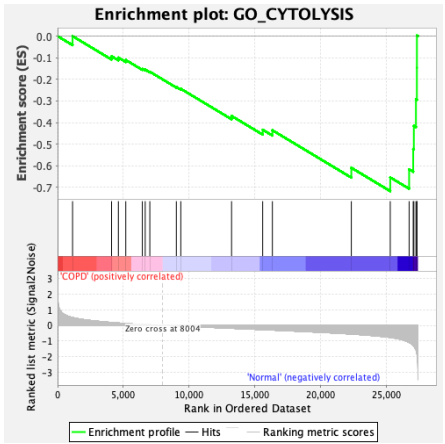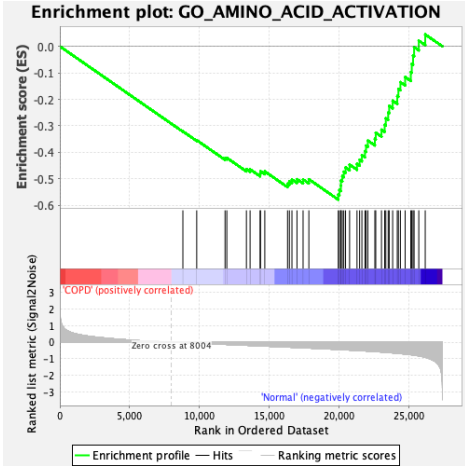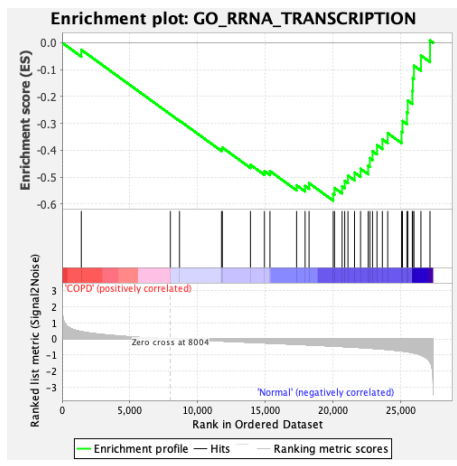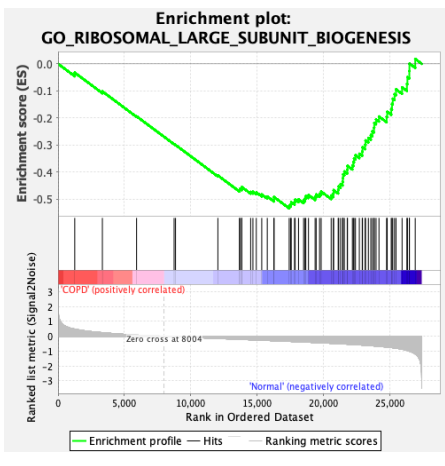

Supplement: Supplementary file 2 — Additional file 2: Fig. S2. GSEA analysis related to COPD from RNA-seq. Enrichment plot of six GO terms associated with COPD pathology, including oxidative phosphorylation, protein folding, cytolysis, amino acid activation, rRNA transcription, ribosomal large subunit biogenesis. [file 12931_2022_2069_MOESM2_ESM.pdf]
