# Supplementary material for: Comprehensive identification of RNA transcripts and construction of RNA network in chronic obstructive pulmonary disease
Source: Respir Res. 2022 Jun 11;23:154. doi: 10.1186/s12931-022-02069-8 (PMC9188256; doi:10.1186/s12931-022-02069-8)
Supplement: Supplementary file 3 — Additional file 3: Table S1. Primers used in this study. [file 12931_2022_2069_MOESM3_ESM.pdf]

Table S1. Primers used in this study

Name	Primer sequence 5' to 3'	Number of bases
circFCHO2-F	TCCAGCTGTGTTGTGCTTCA	20
circFCHO2-R	CAAATAGGGGGTCCCAAGCA	20
circMBOAT2-F	GGATACCTCACAGTGTGCCA	20
circMBOAT2-R	GTTCGAAACCAAATGGCTGCT	21
circPTPN22-F	ACAGACACTGAAGACTCCTGG	21
circPTPN22-R	TGTTCCACCCCATTCCAGTG	20
circTBC1D22A-F	TTCCGCTGGATGAACAACCT	20
circTBC1D22A-R	TCATGCACCAGTAGGTGTCG	20
circACADM-F	ATTCTTTGGGGCAAATGCCTA	21
circACADM-R	ATATTCTGCAGCCACTGGGAT	21
circCKAP5-F	GGAAGCTGGCGATTATGCAG	20
circCKAP5-R	ATCTCGAACAGCCTTCTCTCG	21
CLGN-F	CACTGGGCTGCAGATGGTTG	20
CLGN-R	TATTGGCACTCCTGCTGTCAC	21
GPR42-F	GCAGAGCATTTGGGGTCTCA	20
GPR42-R	CACCGAGAAGACGAACCAGT	20
RSAD2-F	CCTGCTTGGTGCCTGAATCT	20
RSAD2-R	CAGGATGGACTTGGAAGGGTC	21
HLA-DPA1-F	CCCAGGGGACCCTGTGAAAT	20
HLA-DPA1-R	GAGTTTGTAGGGCAGCTGGAG	21
TMEM17-F	ATCTGGGCTACGTGGGTAAC	20
TMEM17-R	CCAAGGGCAGATTTGTTAGGC	21
AC099332.1-F	GGAAACATTCATTGCCACATGC	22
AC099332.1-R	ACATTTCTTGTCCCTGTTCACAC	23
LINC02573-F	TGTGCGTGAATCAAACCAGAG	21
LINC02573-R	ATTGTAAGGTTGCTGGCTGGA	21
AL445493.3-F	CCACAATTGTTGGGAGGATTTGT	23
AL445493.3-R	TGTGTGAACTGGTTATGATTCCA	23
HCG27-F	ACGGAGGCTTTACAAAATAAAATCA	25
HCG27-R	AACACCTGCCTGAGCTTACA	20
AP000465.1-F	TGATTTTCAGGGAGGCATGTTG	22
AP000465.1-R	CAAAGGCTCCCAGTGCCTAC	20
GAPDH-F	CTTCATTGACCTCAACTACATGG	23
GAPDH-R	CTCGCTCCTGGAAGATGGTGAT	22
